# The prevalence of depressive and anxiety symptoms and their associations with quality of life among clinically stable older patients with psychiatric disorders during the COVID-19 pandemic

**DOI:** 10.1038/s41398-021-01196-y

**Published:** 2021-01-26

**Authors:** Wen Li, Na Zhao, Xiaona Yan, Siyun Zou, Huan Wang, Yulong Li, Xiuying Xu, Xiangdong Du, Lan Zhang, Qinge Zhang, Teris Cheung, Gabor S. Ungvari, Chee H. Ng, Yu-Tao Xiang

**Affiliations:** 1 Unit of Psychiatry, Institute of Translational Medicine, Faculty of Health Sciences, University of Macau, Macao SAR, China; 2 Center for Cognition and Brain Sciences, University of Macau, Macao SAR, China; 3 Institute of Advanced Studies in Humanities and Social Sciences, University of Macau, Macao SAR, China; 4 Center for Cognition and Brain Disorders, Institutes of Psychological Sciences, Hangzhou Normal University, Hangzhou, China; 5 Department of Psychiatry, Xiamen Xianyue Hospital, Xiamen, China; 6 Medical College of Soochow University, Suzhou, Jiangsu province China; 7 Guangji Hospital Affiliated to Soochow University, Suzhou, Jiangsu province China; 8 Department of Psychiatry, Lanzhou University Second Hospital, Lanzhou, Gansu province China; 9 The National Clinical Research Center for Mental Disorders & Beijing Key Laboratory of Mental Disorders Beijing Anding Hospital & the Advanced Innovation Center for Human Brain Protection, Capital Medical University, School of Mental Health, Beijing, China; 10 School of Nursing, Hong Kong Polytechnic University, Hong Kong SAR, China; 11 Division of Psychiatry, School of Medicine, University of Western Australia, Perth, WA Australia; 12 University of Notre Dame Australia, Fremantle, WA Australia; 13 Department of Psychiatry, The Melbourne Clinic and St Vincent’s Hospital, University of Melbourne, Richmond, VIC Australia

**Keywords:** Depression, Scientific community

## Abstract

The impact of the COVID-19 pandemic on clinically stable older patients with psychiatric disorders is unclear. This study examined the prevalence of depressive and anxiety symptoms, and their associations with quality of life (QOL) in clinically stable older patients with psychiatric disorders during the COVID-19 pandemic. This was a multicenter, cross-sectional study. Depressive and anxiety symptoms, insomnia, pain, and QOL were assessed with standardized instruments. A total of 1063 patients were included. The prevalence of depressive and anxiety symptoms, and combined depressive and anxiety symptoms were 62.3% (95%CI = 59.4–65.2%), 52.4% (95%CI = 49.3–55.4%), and 45.9% (95%CI = 42.9–48.9%), respectively. Patients with depressive and anxiety symptoms had significantly lower QOL than those without (*P* < 0.01). Binary logistic regression analyses revealed that having depressive symptoms was positively associated with more severe insomnia (OR = 1.29, *P* < 0.01) and pain (OR = 1.14, *P* < 0.01), and was negatively associated with other psychiatric diagnoses (except for major depressive disorder, schizophrenia, and organic mental disorder; OR = 0.50, *P* < 0.01), while having anxiety symptoms was positively associated with severe physical diseases (OR = 1.57, *P* = 0.02), poor adherence to treatment (OR = 1.50, *P* < 0.01), and more severe insomnia (OR = 1.15, *P* < 0.01) and pain (OR = 1.11, *P* < 0.01). Having combined depression and anxiety symptoms was positively associated with poor adherence to treatment (OR = 1.42, *P* = 0.02) and more severe insomnia (OR = 1.19, *P* < 0.01) and pain (OR = 1.15, *P* < 0.01), and was negatively associated with the diagnosis of schizophrenia (OR = 0.50, *P* = 0.04) and others (OR = 0.53, *P* < 0.01). Depressive and anxiety symptoms were common in clinically stable older patients with psychiatric disorders during the COVID-19 pandemic. Considering the negative impact of these symptoms on QOL, regular screening and appropriate treatment are recommended for this population.

## Introduction

As of late July 2020, the Coronavirus Disease 2019 (COVID-19) pandemic has caused ~1 million deaths out of >39 million confirmed cases^1^. Compared to other age groups, older adults are more vulnerable during the COVID-19 pandemic^2^. For instance, older patients with COVID-19 (>50 years) have considerably higher fatality rate^3^ and poorer treatment outcomes^4,5^, which could exacerbate fear and psychological distress due to COVID-19.

Of older adults, those with preexisting psychiatric disorders need greater attention due to the high risk of mental health problems during the COVID-19 pandemic^6,7^. To reduce the risk of disease transmission, some preventive measures, such as quarantine and travel restriction, could trigger or worsen mental health status in older persons with psychiatric problems^7^. For instance, clinically stable older patients with psychiatric disorders usually require long-term maintenance pharmacotherapy. Due to lack of primary mental health services in most areas of China, it is necessary for clinically stable patients to regularly attend psychiatric clinics in urban areas for follow up and medication prescriptions^8,9^. However, as a result of quarantine and travel restriction, patients had difficulties attending hospitals, which may trigger mental health problems, such as depression and anxiety. In addition, older psychiatric patients usually suffer from chronic physical diseases, such as cardiovascular diseases and metabolic disease, which also require long-term medical reviews. Limited access to health services could result in deterioration of their physical diseases and increase their risk of mental health problems.

Depressive and anxiety symptoms (depression and anxiety hereafter) are common mental health problems in older psychiatric patients^10^. These are frequent comorbidities^11,12^ that are associated with poor prognosis^13^, cognitive impairment^14,15^, physical distress^16,17^, and social disability^12^. Even though more attention should be given to depression and anxiety in older psychiatric patients during the COVID-19 pandemic, no relevant studies have been published to date.

A better understanding of the patterns of depression and anxiety is needed to develop appropriate preventive measures and effective treatments for older patients with psychiatric disorders during the COVID-19 pandemic. In addition, quality of life (QOL) is a comprehensive health outcome that reflects individuals’ subjective perception of their overall health status. However, the association of depression and anxiety with QOL in older patients with psychiatric disorders is not clear. Therefore, we conducted this study to examine the prevalence of depression and anxiety, and their associations with QOL among clinically stable older patients with psychiatric disorders during the COVID-19 pandemic. We hypothesized that depression and anxiety were common among clinically stable older patients with psychiatric disorders during the COVID-19 pandemic, and these would have a negative impact on patients’ QOL.

## Methods

### Study sites and participants

This was a cross-sectional study conducted between May 22 and July 15, 2020, in four major tertiary psychiatric hospitals in the eastern (Jiangsu province), southern (Fujian province), western (Gansu province), and northern (Beijing) China, which could represent a wide range of clinical settings in China. Older psychiatric patients receiving maintenance treatments in the outpatient departments of the four hospitals were consecutively invited to participate in the survey, if they met the following criteria: (1) diagnosed with psychiatric disorders according to the International Statistical Classification of Diseases and Related Health Problems, 10th Revision (ICD-10)^18^; (2) older adults aged 50 years and above^19,20^; and (3) clinically stable as assessed by their treating psychiatrists, and able to understand the purpose and procedures of the assessments. Following previous studies^21,22^, “clinically stable patients” were defined as those who had changes in their dose of psychotropic medications of <50% in the past 3 months. This definition was also consistent with the clinical practice in the participating hospitals. Older patients and their guardians (if available) who regularly attended the outpatient clinics for maintenance therapy in each participating hospital were invited by a research psychiatrist with the approval from their treating psychiatrists to participate in this study. After providing their written informed consent on site in the outpatient department of the participating hospitals, they subsequently completed the online assessment using their smartphone. The study protocol was approved by the ethical committees of the participating hospitals.

The sample size was calculated with the following formula^23^: *N* = (*Z*_*α*_^2^ × *P* × (1–*P*))/*d*^2^. The confidence level (*Z*) is equals to 1.96 at significance level of *α* = 0.05, *P* was the estimated proportion, and *d* is the tolerated margin of error and was calculated with 0.1 × *P*. A previous study has found that the prevalence of depression and anxiety were 27.9% and 31.6%, respectively, in the general population^24^. In order to achieve sufficient statistical power, we used *P* = 0.279 to calculate the sample size. Assuming 10% of patients who would refuse the invitation, 1000 patients were needed in this study.

### Data collection and measurements

Due to the potential risk of transmission and logistical reasons during the COVID-19 pandemic, face-to-face interviews could not be performed. Following other studies^25–27^, data were collected using the WeChat-based “Questionnaire Star” program. The WeChat is a social communication program in smartphone with >1 billion users in China. As part of the process of receiving treatments at the participating hospitals, all patients (and/or their guardians) receiving maintenance treatments in the participating outpatient departments were WeChat users. For patients who had difficulties in use of smartphone, the research psychiatrists assisted them to complete the assessment using patients’ or their guardians’ smartphone.

The basic sociodemographic and clinical data (e.g., age, gender, education years, marital status, living area, and presence of severe physical diseases) were collected. Questions with a “yes/no” option were used to collect COVID-19-related data, i.e., “whether or not they were concerned about the COVID-19 pandemic-related information”, “whether or not they frequently used mass media for COVID-19-related information”, “whether or not they had difficulty accessing their psychiatrists during the COVID-19 pandemic”, “whether or not they were adherent to psychiatric treatment during the COVID-19 pandemic”, and “whether or not they had difficulty attending psychiatric hospitals due to the COVID-19 pandemic restrictions”.

Depression was evaluated using the validated Chinese version of the nine-item Patient Health Questionnaire (PHQ-9)^28,29^, which has been widely used in clinical research with a total score ranging from 0 to 27^30^. A PHQ-9 total score of ≥5 was considered as “having depression”, and ≥10 was considered as “having moderate to severe depression”^31^. The severity of anxiety was assessed using the generalized anxiety disorder (GAD-7)^32,33^, with the total score ranging from 0 to 21. A GAD-7 total score of ≥5 was considered as “having anxiety”, and ≥10 was considered as “having moderate to severe anxiety”^32^. Patients with both PHQ-9 total score of ≥5 and GAD-7 total score of ≥5 were considered as “having combined depression and anxiety”.

The severity of insomnia symptoms (insomnia hereafter) was assessed by the seven-item Insomnia Severity Index (ISI)^34^, which has been translated and validated in Chinese populations^35^. The ISI total score ranges from 0 to 28 with a higher total score indicating more severe insomnia. Those with an ISI total score ≥8 were considered “having insomnia symptoms”^36^. The severity of pain was evaluated using the “0–10” numeric rating scale on pain^37,38^, with “0” representing “no pain at all” and “10” representing “unbearable pain”^39^. The overall QOL was measured using the sum of the first two item scores of the World Health Organization Quality of Life-brief version (WHOQOL-BREF)^40–42^, with a higher score representing higher QOL.

### Data analysis

Data were analyzed using the Statistic Package for Social Science (SPSS) version 24.0. The normality of continuous variables was tested, using the P–P plot. The demographic and clinical variables were compared between depression and no depression groups, between anxiety and no anxiety groups, and between combined anxiety and depression and no anxiety or depression groups, respectively. Specifically, *χ*^2^ test was used to compare categorical variables, while two independent samples *t* test and Mann–Whitney *U* test were used to compare normally and non-normally distributed continuous variables, respectively. The association between depression and anxiety were examined with Spearman’s rank-order correlation analysis.

Binary logistic regression analyses with the “enter” method were performed to examine the independent correlates of depression, anxiety, and comorbid anxiety and depression, respectively. All variables with significant group differences in univariate analyses were entered as independent variables, while depression, anxiety, and combined anxiety and depression were entered as the dependent variable separately. The independent associations of depression, anxiety, and comorbid anxiety and depression with QOL were examined using analysis of covariance (ANCOVA) after controlling for variables with significant group differences in univariate analyses. The significance level was set at *P* < 0.05 (two-tailed).

## Results

### Social-demographic and clinical characteristics

Altogether, 1068 patients were invited to participate in the survey during the predefined study period. Finally, 1063 met the eligibility criteria and were included, giving a response rate of 99.5%. The sociodemographic and clinical characteristics of the participants were presented in Table 1. The mean age of participants was 62.8 (standardized deviation (SD) = 9.4) years and 32.6% (*n* = 347) of the total sample were males.

**Table 1:** The sociodemographic and clinical characteristics.

Variable	Total (*N* = 1063)	No DEP (*N* = 400)	DEP (*N* = 663)	Univariate analyses			No ANX (*N* = 506)	ANX (*N* = 557)	Univariate analyses			No DEP or ANX (*N* = 575)	Comorbid DEP and ANX (*N* = 488)	Univariate analyses		
	*N* (%)	*N* (%)	*N* (%)	*χ*^2^	d.f.	*P*	*N* (%)	*N* (%)	*χ*^2^	d.f.	*P*	*N* (%)	*N* (%)	*χ*^2^	d.f.	*P*
Male gender	347 (32.6)	126 (31.5)	221 (33.3)	0.38	1	0.53	163 (32.2)	184 (33.0)	0.08	1	0.77	188 (32.7)	159 (32.6)	0.00	1	0.96
Married	961 (90.4)	364 (91.0)	597 (90.0)	0.26	1	0.60	461 (91.1)	500 (89.8)	0.54	1	0.45	524 (91.1)	437 (89.5)	0.76	1	0.38
Rural areas	373 (35.1)	111 (27.8)	262 (39.5)	15.16	1	**<0.01**	151 (29.8)	222 (39.9)	11.67	1	**<0.01**	169 (29.4)	204 (41.8)	17.85	1	**<0.01**
Having severe physical diseases	190 (17.9)	59 (14.8)	131 (19.8)	4.26	1	**0.03**	69 (13.6)	121 (21.7)	11.81	1	**<0.01**	84 (14.6)	106 (21.7)	9.09	1	**<0.01**
Concerns about COVID-19	744 (70.0)	274 (68.5)	470 (70.9)	0.67	1	0.41	358 (70.8)	386 (69.3)	0.26	1	0.60	404 (70.3)	340 (69.7)	0.04	1	0.83
Frequent use of mass media^a^	215 (20.2)	89 (22.3)	126 (19.0)	1.62	1	0.20	102 (20.2)	113 (20.3)	0.00	1	0.95	117 (20.3)	98 (20.1)	0.01	1	0.91
Difficulty accessing psychiatrists^a^	367 (34.5)	131 (32.8)	236 (35.6)	0.89	1	0.34	163 (32.2)	204 (36.6)	2.28	1	0.13	190 (33.0)	177 (36.3)	1.21	1	0.27
Poor treatment adherence^a^	365 (34.3)	98 (24.5)	267 (40.3)	27.52	1	**<0.01**	131 (25.9)	234 (42.0)	30.56	1	**<0.01**	156 (27.1)	209 (42.8)	28.84	1	**<0.01**
Difficulty attending psychiatric hospital^a^	371 (34.9)	113 (28.3)	258 (38.9)	12.48	1	**<0.01**	157 (31.0)	214 (38.4)	6.37	1	**0.01**	179 (31.1)	192 (39.3)	7.83	1	**<0.01**
Primary psychiatric diagnoses				18.01	3	**<0.01**			16.59	3	**<0.01**			30.34	3	**<0.01**
Major depressive disorder	485 (45.6)	152 (38.0)	333 (50.2)				207 (40.9)	278 (49.9)				225 (39.1)	260 (53.3)			
Schizophrenia	73 (6.9)	38 (9.5)	35 (5.3)				48 (9.5)	25 (4.5)				55 (9.6)	18 (3.7)			
Organic mental disorder	63 (5.9)	26 (6.5)	37 (5.6)				27 (5.3)	36 (6.5)				32 (5.6)	31 (6.4)			
Others^b^	442 (41.6)	184 (46.0)	258 (38.9)				224 (44.3)	218 (39.1)				263 (45.7)	179 (36.7)			

	*M* (SD)	*M* (SD)	*M* (SD)	*t/Z*	d.f.	*P*	*M* (SD)	*M* (SD)	*t/Z*	d.f.	*P*	*M* (SD)	*M* (SD)	*t/Z*	d.f.	*P*
Age (years)	62.8 (9.4)	63.5 (9.6)	62.3 (9.2)	1.90	1061	0.05	62.8 (9.3)	62.7 (9.5)	0.16	1061	0.87	63.1 (9.4)	62.4 (9.3)	1.19	1061	0.23
Education years	7.9 (4.0)	8.3 (3.6)	7.7 (4.2)	2.20	1061	**0.02**	8.1 (3.6)	7.7 (4.3)	1.74	1061	0.08	8.1 (3.6)	7.7 (4.4)	1.38	1061	0.16
ISI total score	8.9 (6.3)	4.5 (4.3)	11.5 (5.9)	−17.87	—^c^	**<0.01**	6.2 (5.3)	11.3 (6.2)	−13.09	—^c^	**<0.01**	6.2 (5.3)	12.1 (6.0)	−15.15	—^c^	**<0.01**
Pain score	1.5 (2.2)	0.9 (1.5)	1.9 (2.4)	−6.47	—^c^	**<0.01**	0.9 (1.6)	2.0 (2.5)	−6.88	—^c^	**<0.01**	0.9 (1.6)	2.2 (2.5)	−8.09	—^c^	**<0.01**
Quality of life	6.2 (1.5)	7.0 (1.2)	5.7 (1.5)	13.41	1061	**<0.01**	6.8 (1.3)	5.7 (1.5)	12.48	1061	**<0.01**	6.8 (1.3)	5.5(1.5)	14.05	1061	**<0.01**

Values in bold indicate <0.05.

*ANX* anxiety, *COVID-19* Coronavirus Disease 2019, *DEP* depression, *ISI* Insomnia Severity Index, *M* mean, *SD* standard deviation.

^a^Observation period was during the COVID-19 pandemic.

^b^Only psychiatric diagnoses with the percentage of >5% are presented separately; those with percentage of <5% were included in “others”.

^c^Mann–Whitney *U* tests.

The prevalence of the overall depression (PHQ total score ≥5) was 62.3% (95%CI = 59.4–65.2%), while the prevalence of moderate to severe depression (PHQ total score ≥10) was 30.8% (95%CI = 28.0–33.6%). The mean total score of PHQ-9 was 7.75 (SD = 6.73). The prevalence of the overall anxiety (GAD-7 total score ≥5) was 52.4% (95%CI = 49.3–55.4%), while the prevalence of moderate to severe anxiety (GAD-7 total score ≥10) was 25.6% (95%CI = 23.0–28.3%). The mean total score of GAD-7 was 5.95 (SD = 5.76). Spearman’s rank-order correlation analysis revealed that depression and anxiety had a significant correlation (correlation coefficient = 0.73, *P* < 0.01). The prevalence of combined depression and anxiety was 45.9% (95%CI = 42.9–48.9%). The prevalence of insomnia (ISI total score ≥8) was 56.9% (95%CI = 53.9–59.9%).

### Univariate analyses

Univariate analyses revealed that patients with depression were more likely to live in rural areas (*P* < 0.01), suffer from severe physical diseases (*P* = 0.03), and have lower education level (*P* = 0.02), poor treatment adherence (*P* < 0.01), and difficulty attending psychiatric hospitals (*P* < 0.01) during the COVID-19 pandemic, and suffer from more severe insomnia (*P* < 0.01) and pain (*P* < 0.01). The prevalence of depression was significantly different across different psychiatric diagnoses (*P* < 0.01) (Table 1).

Patients with anxiety were more likely to live in rural area (*P* < 0.01), suffer from severe physical diseases (*P* < 0.01), have poor treatment adherence (*P* < 0.01), and difficulty attending psychiatric hospitals (*P* = 0.01) during the COVID-19 pandemic, and have more severe insomnia (*P* < 0.01) and pain (*P* < 0.01). The prevalence of anxiety was significantly different across different psychiatric diagnoses (*P* < 0.01) (Table 1).

Patients with combined depression and anxiety were more likely to live in rural areas (*P* < 0.01), suffer from severe physical diseases (*P* < 0.01), have poor treatment adherence (*P* < 0.01), and difficulty attending psychiatric hospitals (*P* < 0.01) during the COVID-19 pandemic, and have more severe insomnia (*P* < 0.01) and pain (*P* < 0.01). The prevalence of combined depressive and anxiety symptoms was significantly different across different psychiatric diagnoses (*P* < 0.01) (Table 1).

### Multivariate analyses

Table 2 presents the results of binary logistic regression analyses. Depression was positively associated with more severe insomnia (OR = 1.29, 95%CI = 1.24–1.34, *P* < 0.01) and pain (OR = 1.14, 95%CI = 1.03–1.25, *P* < 0.01), and was negatively associated with other psychiatric diagnoses (except for major depressive disorder, schizophrenia, and organic mental disorder; OR = 0.50, 95%CI = 0.35–0.71, *P* < 0.01). Anxiety was positively associated with severe physical diseases (OR = 1.57, 95%CI = 1.05–2.35, *P* = 0.02), poor adherence to treatment (OR = 1.50, 95%CI = 1.11–2.03, *P* < 0.01), and more severe insomnia (OR = 1.15, 95%CI = 1.12–1.18, *P* < 0.01) and pain (OR = 1.11, 95%CI = 1.02–1.20, *P* < 0.01). Combined depression and anxiety were positively associated with poor adherence to treatment (OR = 1.42, 95%CI = 1.03–1.95, *P* = 0.02), and more severe insomnia (OR = 1.19, 95%CI = 1.16–1.23, *P* < 0.01) and pain (OR = 1.15, 95%CI = 1.06–1.25, *P* < 0.01), and was negatively associated with schizophrenia (OR = 0.50, 95%CI = 0.26–0.97, *P* = 0.04) and other psychiatric diagnoses (except for major depressive disorder, schizophrenia, and organic mental disorder; OR = 0.53, 95%CI = 0.38–0.73, *P* < 0.01) (Table 2).

**Table 2:** Independent correlates of depression, anxiety, and combined depression and anxiety in older patients with psychiatric disorder.

Variable	Depression^a^			Anxiety^a^			Combined depression and anxiety^a^		
	*P*	OR	95%CI	*P*	OR	95%CI	*P*	OR	95%CI
Rural area	0.18	1.29	0.88–1.89	0.27	1.20	0.86–1.68	0.08	1.36	0.95–1.93
Having severe physical diseases	0.20	1.35	0.85–2.15	**0.02**	1.57	1.05–2.35	0.05	1.49	0.98–2.26
Poor treatment adherence^b^	0.20	1.25	0.88–1.78	**<0.01**	1.50	1.11–2.03	**0.02**	1.42	1.03–1.95
Difficulty attending psychiatric hospital^b^	0.08	1.38	0.95–1.99	0.08	1.33	0.96–1.84	0.06	1.37	0.97–1.91
Primary psychiatric diagnoses									
Major depressive disorder	Ref.	—	—	Ref.	—	—	Ref.	—	—
Schizophrenia	0.88	0.95	0.51–1.77	0.19	0.67	0.37–1.22	**0.04**	0.50	0.26–0.97
Organic mental disorder	0.12	0.57	0.28–1.16	0.44	0.78	0.41–1.47	0.23	0.66	0.34–1.29
Others^c^	**<0.01**	0.50	0.35–0.71	0.06	0.74	0.54–1.01	**<0.01**	0.53	0.38–0.73
Education years	0.67	0.99	0.94–1.03	0.25	0.97	0.93–1.01	0.61	0.98	0.94–1.03
ISI total score	**<0.01**	1.29	1.24–1.34	**<0.01**	1.15	1.12–1.18	**<0.01**	1.19	1.16–1.23
Pain score	**<0.01**	1.14	1.03–1.25	**<0.01**	1.11	1.02–1.20	**<0.01**	1.15	1.06–1.25

Values in bold indicate <0.05.

*CI* confidential interval, *COVID-19* Coronavirus Disease 2019, *ISI* Insomnia Severity Index, *OR* odds ratio, *Ref.* reference group.

^a^Residences were controlled as covariate.

^b^Observation period was during the COVID-19 outbreak.

^c^Only psychiatric diagnoses with the percentage of >5% are presented separately; those with percentage of <5% were included in “others”.

ANCOVA revealed that patients with depression had significantly lower overall QOL compared to those without depression (*F*_(1, 1063)_ = 36.11, *P* < 0.01), while patients with anxiety had significantly lower overall QOL compared to those without anxiety (*F*_(1, 1063)_ = 40.35, *P* < 0.01), and patients with combined depression and anxiety had significantly lower overall QOL compared to those without depression and anxiety (*F*_(1, 1063)_ = 51.85, *P* < 0.01).

## Discussion

This was the first study that examined depression and anxiety in clinically stable older patients with psychiatric disorders during a pandemic. The prevalence of depression (62.3%) and anxiety (52.4%) in this study were higher than the corresponding figures in adolescents (depression: 43.7%; anxiety: 37.4%)^43^ and in the general population (depression: 27.9%; anxiety: 31.6%%)^24^ in China, using the same measurements during the COVID-19 pandemic. This high prevalence of depression and anxiety in older psychiatric patients could be partly due to the fear of high mortality rate and poor prognosis in older people infected with COVID-19^3–5^, which leads to considerable psychological distress in older population. Furthermore, many older psychiatric patients suffer from comorbid impaired cognitive function^44–47^, which may cause difficulties to understand and process COVID-19-related information, resulting in depression and anxiety^48^. In addition, the quarantine and travel restrictions during the COVID-19 pandemic did not only create barriers to access health services, but also cause disruptions to regular daily life and activities, all of which could increase the risk of depression and anxiety^49^.

In this study, comorbid depression and anxiety were not only common (45.9%, 95%CI = 42.9–48.9%) among participants, but also significantly correlated with each other, which is consistent with previous findings^11,13,50^. The association between depression and anxiety is bidirectional; anxiety can trigger depression^51^ and vice versa^52^. The co-occurrence of depression and anxiety may be related to alteration of activation and connectivity of ventral cingulate and amygdala, and the polymorphic variations in the serotonin receptor gene^53,54^.

Patients with depression and/or anxiety reported more severe insomnia. The association between insomnia and psychiatric disorders is complex. On the one hand, insomnia can increase the risk of psychiatric disorders, including depression and anxiety^55,56^. For instance, a meta-analysis found that insomnia is a major risk factor of depression (OR = 2.83, 95%CI = 1.55–5.17) and anxiety (OR = 3.23, 95%CI = 1.52–6.85)^56^. On the other hand, depression and anxiety can also contribute to the development of insomnia^57,58^. Comorbid insomnia and psychiatric disorders may share common pathophysiology, such as similar alterations of arousal states^59^ and levels of inflammatory markers^60^.

Depression and anxiety were associated with more severe pain in this study. Chronic pain (e.g., headache and musculoskeletal pain) is common in older population^61^, and has a negative impact on QOL, sleep quality, and social functioning^62^. Previous studies found that certain psychiatric disorders (e.g., depression and anxiety) and pain commonly coexisted^63^, with bidirectional associations^64–67^.

We found that anxiety and combined depression and anxiety was positively associated with poor treatment adherence, which is consistent with previous findings that patients with anxiety were more likely to discontinue their medication treatment due to side effects^68^, or their psychological interventions due to low motivation and poor therapeutic alliance^69^. The positive association between anxiety and comorbid severe physical diseases (e.g., cardiac vascular diseases, gastrointestinal diseases, and genitourinary disorders) could be due to relevant to various factors, including physical distress, medication-induced side effects, and treatment cost^16,70–72^. In this study, patients with schizophrenia and other psychiatric disorders, except for major depressive disorder and organic mental disorder, were less likely to have combined depressive and anxiety symptoms, while patients with other psychiatric disorders, except for major depressive disorder and organic mental disorder, were less likely to have depressive symptoms alone during the COVID-19 pandemic. However, it is noteworthy that there were only 73 schizophrenia patients and 63 patients with organic mental disorders; therefore, the associations between primary psychiatric diagnoses and psychiatric symptoms during the COVID-19 pandemic needs to be confirmed in future head-to-head comparative studies.

As expected, patients with depression and anxiety had lower QOL than those without. QOL is determined by the interaction between protective factors (e.g., better social support and higher financial level) and distressing factors (e.g., poor mental health and physical conditions)^73^. Depression and anxiety were associated with cognitive dysfunction^14,15^, physical distress^16,17^, and impaired social functioning^12^, which could lower patients’ QOL.

The strengths of this study included the large sample size, consecutive sampling, high participation rate, and multicenter study design. However, several limitations need to be addressed. First, this study only recruited clinically stable patients from outpatient departments, which could limit the generalization of the findings to those in acute illness phase. Second, due to the cross-sectional study design, the causal relationships between depression/anxiety and other variables could not be examined. Third, some factors associated with depression and anxiety, such as cognitive function^15,74^, and type and dose of psychotropic medications^17^, were not assessed in this study due to logistical reasons. Fourth, COVID-19-related information was assessed with self-reported standardized questions. The results of relevant objective measures would be more reliable, but they were not developed at the study time.

In conclusion, depression and anxiety were common in clinically stable older patients with psychiatric disorders. Considering the negative outcomes caused by depression and anxiety, regular screening, and effective treatment, such as appropriate pharmacotherapy and psychotherapy (e.g., cognitive behavior therapy)^75,76^, should be recommended for this population.
